# People punish defection, not failures to conform to the majority

**DOI:** 10.1038/s41598-023-50414-8

**Published:** 2024-01-12

**Authors:** Ana Philippsen, Laura Mieth, Axel Buchner, Raoul Bell

**Affiliations:** Department of Experimental Psychology, Heinrich Heine University Düsseldorf, Universitätsstrasse 1, 40225 Düsseldorf, Germany

**Keywords:** Psychology, Human behaviour

## Abstract

Do people punish others for defecting or for failing to conform to the majority? In two experiments, we manipulated whether the participants’ partners cooperated or defected in the majority of the trials of a Prisoner’s Dilemma game. The effects of this base-rate manipulation on cooperation and punishment were assessed using a multinomial processing tree model. High compared to low cooperation rates of the partners increased participants’ cooperation. When participants’ cooperation was not enforced through partner punishment, the participants’ cooperation was closely aligned to the cooperation rates of the partners. Moral punishment of defection increased when cooperation rates were high compared to when defection rates were high. However, antisocial punishment of cooperation when defection rates were high was much less likely than moral punishment of defection when cooperation rates were high. In addition, antisocial punishment was increased when cooperation rates were high compared to when defection rates were high. The latter two results contradict the assumption that people punish conformity-violating behavior regardless of whether the behavior supports or disrupts cooperation. Punishment is thus sensitive to the rates of cooperation and defection but, overall, the results are inconsistent with the idea that punishment primarily, let alone exclusively, serves to enforce conformity with the majority.

The capacity for large-scale cooperation has crucially fostered human evolution and the establishment of societies as we know them today. As cooperation implies accepting personal costs for achieving a long-term collective benefit, there is often an incentive to free ride on the other’s cooperation. This clash of individual and collective interests creates a social dilemma [cf.^1^]. The free-rider problem poses a threat: If too many people free ride, cooperation continuously loses its appeal, declines and the system collapses^2–5^. Cooperation levels vary strongly between groups as a function of a number of different factors and may fall above or below 50%, depending on the situation^6,7^. One factor that is often believed to support the maintenance of cooperation is the punishment of people who refuse to cooperate and instead defect^8–10^. While punishment of defection in repeated interactions can obviously benefit the punishing individuals by enforcing cooperation of their partners in future interactions, punishment of defection in one-shot interactions is more challenging to explain. Irrespective of this, it is a fact that people punish defectors even in one-shot interactions in which there are no obvious incentives for doing so. This is evident not only in the lab^11–13^ but also in everyday social interactions. For example, in a one-time interaction on an online shopping site, buyers who feel they were treated unfairly (e.g., because they ordered goods that later turn out to be of poorer quality than advertised) may spend time and effort to write negative reviews to punish the seller. It is thus important to gain a better understanding of this puzzling yet socially tangible behavior. Two possible explanations can be distinguished for why people punish defection in one-shot interactions. One possibility is that cooperating individuals punish others specifically for their defection^14^. Another possibility is that people punish behavior to enforce conformity with the majority regardless of whether it supports or disrupts cooperation^15–18^. Here we test these accounts by examining how a manipulation of the proportions of cooperation and defection affects costly punishment in a Prisoner’s Dilemma game.

The Prisoner’s Dilemma game is a classical paradigm for studying cooperation. In this game, two players simultaneously decide to either cooperate or defect which leads to different possible outcomes, as determined by the game’s payoff structure (see Fig. 1). A defecting player who interacts with a cooperating partner receives the highest outcome. A cooperating player who interacts with a defecting partner receives the lowest outcome. At an individual level, it is therefore more profitable to defect. At a collective level, however, cooperation is desirable because mutual cooperation leads to a better outcome for both interactants combined than mutual defection. This payoff structure thereby captures the basic dilemma of cooperation [cf.^1^].

**Figure 1:**
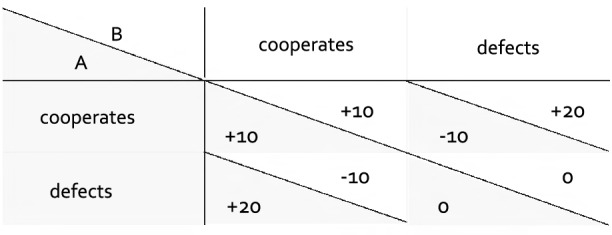
Examples of a payoff structure of the Prisoner’s Dilemma game. The payoffs are displayed as a function of both players’ decisions in the Prisoner’s Dilemma game. Shaded cells denote the payoff to Player A, white cells denote the payoff to Player B.

People often strive to achieve mutual cooperation but try to avoid being cheated by a defecting partner who does not reciprocate cooperation. Therefore, it comes as no surprise that cooperation in economic dilemmas is often conditioned on the perceived or proclaimed prevalence of cooperation^16,19–23^. For example, Engel et al.^23^ provided their participants with selective information about the behavior of either very cooperative or very uncooperative groups before participating in an economic game. Participants were more likely to cooperate when they had received information about the behavior of cooperative groups than when they had received information about the behavior of uncooperative groups. These findings indicate that a person’s propensity to cooperate is influenced by the assumed prevalence of cooperation.

A factor that has been shown to crucially contribute to the maintenance of cooperation within groups is moral punishment [cf.^2^]. Here, the term *moral punishment* is used to specifically refer to the punishment of defecting partners by cooperating individuals. Defection becomes unattractive when a significant proportion of people punish defection because punishment decreases the payoffs of defecting partners. Moral punishment can thus help to solve the free-rider problem by disincentivizing defection, thereby increasing the level of cooperation^8,11,13,24,25^. However, moral punishment often entails personal costs to the punisher. Therefore, moral punishment can be considered a second-order cooperative act^5,11,13,26,27^. Given the importance of moral punishment for the establishment and maintenance of cooperation, it is crucial to understand the factors that drive people to punish others for defection.

Two broad accounts can be distinguished with regard to how the proportion of cooperation or defection should affect people’s punishment behavior. One possibility is that punishment is primarily used to discourage defection, regardless of the prevalence of defection [e.g.,^14^]. This seems reasonable as punishment in economic games is, as a rule, mainly directed at defectors. However, in a small proportion of cases, people may prefer not to cooperate, sometimes leading to *antisocial punishment* of cooperative acts^28–30^. This type of punishment is termed antisocial as it undermines cooperation^31,32^. A possible explanation is that antisocial punishers are motivated by their disapproval of the normative pressure towards cooperation, exerted by individuals who are perceived as moral “do-gooders”^33–35^. While it may, at first glance, seem obvious that people should punish behaviors they disapprove of—which would explain the prevalence of both moral *and* antisocial punishment—it has been suggested that people do at least sometimes punish others for failing to conform to the majority regardless of their own private preferences^36^. The *conformity account* implies that punishment is directed at behavior that deviates from what is typical^15–18,36^. People may punish atypical behaviors to enforce conformity as conformity may reduce the costs that result from conflicts arising from uncertainty about the appropriate behavior. Furthermore, people may engage in punishment when they think that the punishment is justified by the fact that others approve of their punishment which also keeps the costs of punishment low^17^. Considering the high prevalence of cooperation in human groups and societies, punishment will often be directed at defectors who fail to contribute to the collective benefit. However, there is a dark side to enforcing conformity irrespective of the consequences of the behavior: People may antisocially punish atypical behavior even when it is promoting the collective good^16,17^ simply because it violates expectations.

Here we examine how the proportion of cooperation and defection affects costly punishment in the Prisoner’s Dilemma game. This study follows a previous study by Li et al.^37^ in which participants had to decide between cooperation and defection in a Prisoner’s Dilemma game. Prior to making their punishment decision, participants received information about eleven possible scenarios regarding how many other players had previously chosen to cooperate (ranging from less than 5% to more than 95%). Each participant was then asked to make a punishment decision for defecting partners in every one of these hypothetical scenarios. Punishment increased with the percent of cooperation in the reference group. Apart from the fact that conceptual replications of important findings are always useful, there are several additional reasons to expand on the previous findings. First, Li et al.^37^ asked participants to respond to a list of eleven scenarios with different hypothetical base rates which may have accentuated the impact of the base rates on behavior. It is thus interesting to examine whether moral punishment increases with the proportion of cooperation when participants interact with partners directly. Second, Li et al.^37^ concentrated only on moral punishment by requiring participants to provide punishment decisions only for defecting partners. Here, we allow participants to make punishment decisions regardless of the outcome of the Prisoner’s Dilemma game which gives us the opportunity to distinguish between different types of punishment.

To allow to cleanly distinguish between different types of punishment and a bias towards punishing, the *multinomial cooperation-and-punishment model* has been developed. The model belongs to the class of multinomial processing tree models. These models have become increasingly popular to measure the components of human decision making [for a review see^38^]. Multinomial models are flexible and accessible measurement models for which easy-to-read tutorials^39^ and user-friendly software^40^ exists. They disambiguate observable behavior by enabling the measurement of the processes underlying overt behavior such as different strategies in decision-making tasks^41–43^. The relationship between observable behavioral categories and the underlying processes can be visualized in a tree-like structure. Here, we use the multinomial cooperation-and-punishment model (see Fig. 3) which has been successfully applied and validated in previous studies^44–46^. Besides the cooperation parameter *C*, representing the participants’ propensity to cooperate, the model entails that specific types of punishment have to be distinguished from a general punishment bias. *Moral punishment* is defined as the type of punishment that is specifically provoked when the participant’s cooperation is met with the partner’s defection. This type of punishment can be viewed as moral because it is aimed at retaliating the perceived violation of a cooperation norm. To illustrate, moral punishment is enhanced when the labels of the behavioral options in the Prisoner’s Dilemma game facilitate a moral interpretation of the behaviors relative to when the labels are neutral^44^. *Hypocritical punishment* is the type of punishment that is specifically provoked by an interaction in which both the participant and the partner chose to defect. This type of punishment can be viewed as hypocritical because participants punish behavior in others which they themselves have shown. *Antisocial punishment* is specifically provoked by an interaction in which the participant’s defection is met with a partner’s cooperation. This type of punishment can be labeled as antisocial in the sense that it reflects an opposition against cooperation norms. To illustrate, previous studies^44,46^ have shown that antisocial punishment is increased when participants experience normative pressure to cooperate through the moral punishment exerted by the partners. Furthermore, a proper measurement model of punishment has to take an unspecific bias to punish into account. This allows us to test whether the observed effects are distinct for the different punishment types or reflect a general increase in the willingness to punish, for example, as a way to vent frustration about factors that are unrelated to the outcome of the immediate interaction^46^.

To test whether people primarily punish others for violating conformity, we manipulated the proportion of cooperating and defecting partners in the Prisoner’s Dilemma game between groups. In the *cooperating-majority condition*, partners cooperated in 60% of the trials and defected in the other 40%, thereby making cooperation the dominant behavior. In the *defecting-majority condition*, this ratio was reversed, thereby making defection the dominant behavior. To ensure that the behavior of the majority was correctly represented, the participants were truthfully informed prior to the start of the game whether most partners would cooperate or defect. If punishment primarily serves to discourage defection, moral punishment should prevail irrespective of the base-rate manipulation. If punishment primarily serves to enforce conformity, punishment should be highly susceptible to the base-rate manipulation. Specifically, moral punishment should be high in the cooperating-majority condition but low or even absent in the defecting-majority condition^37^. Based on the idea that people may enforce conformity with the majority behavior regardless of their own preferences^36^, hypocritical punishment should follow the same pattern as moral punishment. Hypocritical punishment should thus be increased in the cooperating-majority condition in comparison to the defecting-majority condition. If people punish to enforce conformity with the dominant behavior in the Prisoner’s Dilemma game, antisocial punishment, that is, the punishment of cooperation by defecting participants, should be high in the defecting-majority condition but low or even absent in the cooperating-majority condition^16,17^. In fact, if punishment were exclusively determined by the goal to enforce conformity, then the probability that cooperating participants use moral punishment to punish a deviation from a cooperating majority should be identical to the probability that defecting participants use antisocial punishment to punish a deviation from a defecting majority.

## Experiment 1

### Method

#### Sample

We aimed to obtain about 500 valid data sets in each of the two experiments with the help of the online panel provider *mingle*. Of the data files of those participants who started the Prisoner’s Dilemma game, 54 data files had to be removed because the participants did not complete the experiment and 70 data files had to be excluded due to double participation. The final sample consisted of 544 participants (305 female, 239 male) aged 18–88 (*M* = 49, *SD* = 15) years. A sensitivity analysis showed that with a sample size of *N* = 544 and 25 decisions per participant it was possible to detect effects of the base-rate manipulation on the cooperation and punishment parameters of the multinomial cooperation-and-punishment model (see below) of the size *w* = 0.03 with a statistical power of 1 − β = 0.95 at an α level of 0.05^47^.

#### Base-rate manipulation

At the start of the experiment, participants were assigned to either the cooperating-majority condition (*n* = 278) or the defecting-majority condition (*n* = 266). Depending on the assigned condition, participants were instructed either that most people would cooperate and only some would defect or that most people would defect and only some would cooperate. These instructions were used to ensure that participants formed a correct representation about the majority behavior even before the Prisoner’s Dilemma game started.

The fact that the partners’ responses were determined by a computer program then allowed us to manipulate the proportion of cooperating and defecting partners in line with these instructions. Experimentally manipulating the partner behavior is a common approach in Experimental Psychology to generate varying base rates while maintaining control over confounding factors that may otherwise influence partner behavior^16,48–54^. In the cooperating-majority condition, partners were programmed to cooperate in 60% of the trials and to defect in 40% of the trials. In the defecting-majority condition, this ratio was reversed.

#### Prisoner’s Dilemma game

Materials and procedure of the Prisoner’s Dilemma game were parallel to those of a previous online study examining costly punishment in the Prisoner’s Dilemma game^46^. After giving their informed consent and answering demographic questions, participants received the instructions for the Prisoner’s Dilemma game. Participants of the online panel provider mingle are compensated with points that can be exchanged for online vouchers, charity donations or money (with 1 point corresponding to 1 Euro cent). Participants were thus informed that they were playing for points which they would be awarded by mingle at the end of the study in addition to the points they would receive for participating in the study. At the start of the experiment, participants were endowed with 150 points. Participants played 30 trials, five of which were training trials, of a simultaneous one-shot Prisoner’s Dilemma game with a costly punishment option.

**Figure 2:**
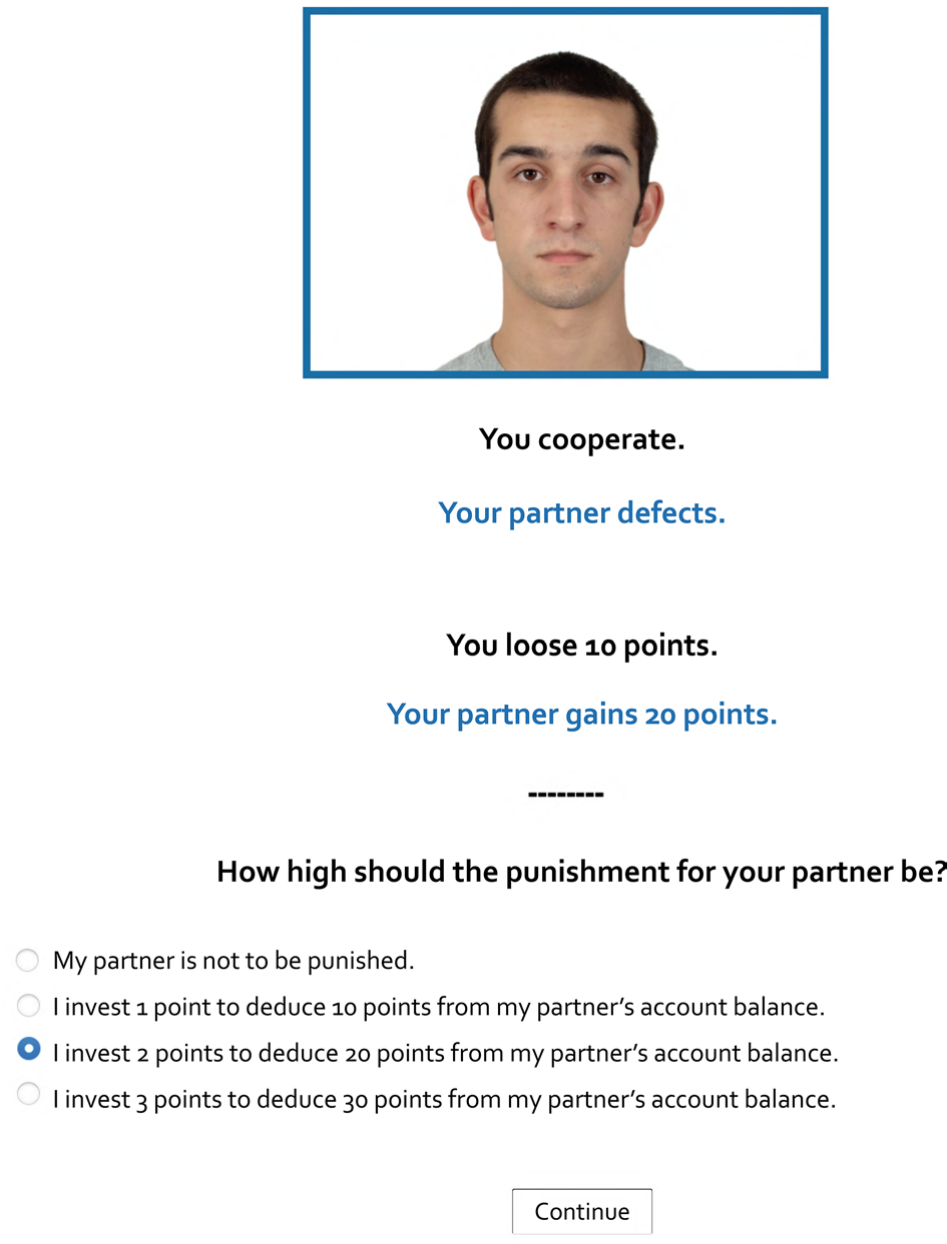
Example trial of the Prisoner’s Dilemma game with costly punishment. In this example trial, the participant cooperated while the partner defected which led to a loss of 10 points for the participant and a gain of 20 points for the partner. The participant then chose to morally punish the partner by investing 2 points so that 20 points were subtracted from the partner’s account balance. The partner’s photograph was randomly selected from the Chicago Face Database^55^.

Each trial of the Prisoner’s Dilemma started with the display of the participant’s current account balance in the middle of the screen. Participants knew that they would interact with a different partner in every trial. Upon clicking a “Continue” button, the interaction partner was shown. To emphasize the social nature of the game, participants saw a color photograph (266 × 186 pixels) of a different partner in each trial. To this end, photographs of 30 white adult faces, half of which were female and half of which were male, were randomly drawn from the Chicago Face Database^55^. All faces had a neutral expression and were shown from a frontal view. The partner’s photograph was centered on-screen and surrounded by a blue frame (4 pixels, see Fig. 2).

Beneath the photograph, participants could choose to cooperate or to defect by clicking the corresponding button and submitting their choice with a “Continue” button. Participants had been instructed that they and their partner would see their decisions to cooperate or to defect simultaneously. There were four different outcomes depending on both partners’ decisions, as illustrated by the payoff matrix in Fig. 1. Participants knew that mutual cooperation would lead to a gain of 10 points for each partner while mutual defection would lead to no gain or loss. They also knew that a defecting partner would gain 20 points when interacting with a cooperating partner who would in return lose 10 points. Participants received feedback about their own decision (e.g., “You cooperate.”) and their partner’s decision (e.g., “Your partner defects.”) and how these decisions affected each players’ account balance (e.g., “You lose 10 points.”, “Your partner gains 20 points.”). Feedback regarding the participant’s decision and outcome was displayed in black font color whereas feedback on the partner’s decision and outcome was shown in blue font color, corresponding to the blue frame around the partner’s photograph. The photograph and the feedback of the interaction outcome remained visible on the screen until the end of each trial.

#### Costly-punishment option

After each interaction in the Prisoner’s Dilemma, participants were offered a costly punishment option. Participants could decide either not to punish their partner or to invest 1, 2 or 3 points to deduce 10, 20 or 30 points, respectively, from their partner’s account balance. Participants were informed beforehand that their partners would simultaneously make their decision to punish the participants. As in a previous experiment^46^, the partners were programmed to always punish unilateral defection of the participants by deducing a randomly determined amount of 10, 20 or 30 points from the participants’ account. Upon clicking a “Continue” button, participants received feedback about their own punishment decision (e.g., “You invest 2 points to punish your partner.”) and its effect on the partner’s account balance (e.g., “20 points will be deducted from your partner’s account balance.”). Participants simultaneously learned about their partner’s punishment decision (e.g., “Your partner does not punish you.”) and its effect on their own account balance (e.g., “No fine will be deducted from your account balance.”). With a “Continue” button, participants could then start the next trial. The average final account balance was 128 (*SD* = 54) points.

#### The cooperation-and-punishment model

**Figure 3:**
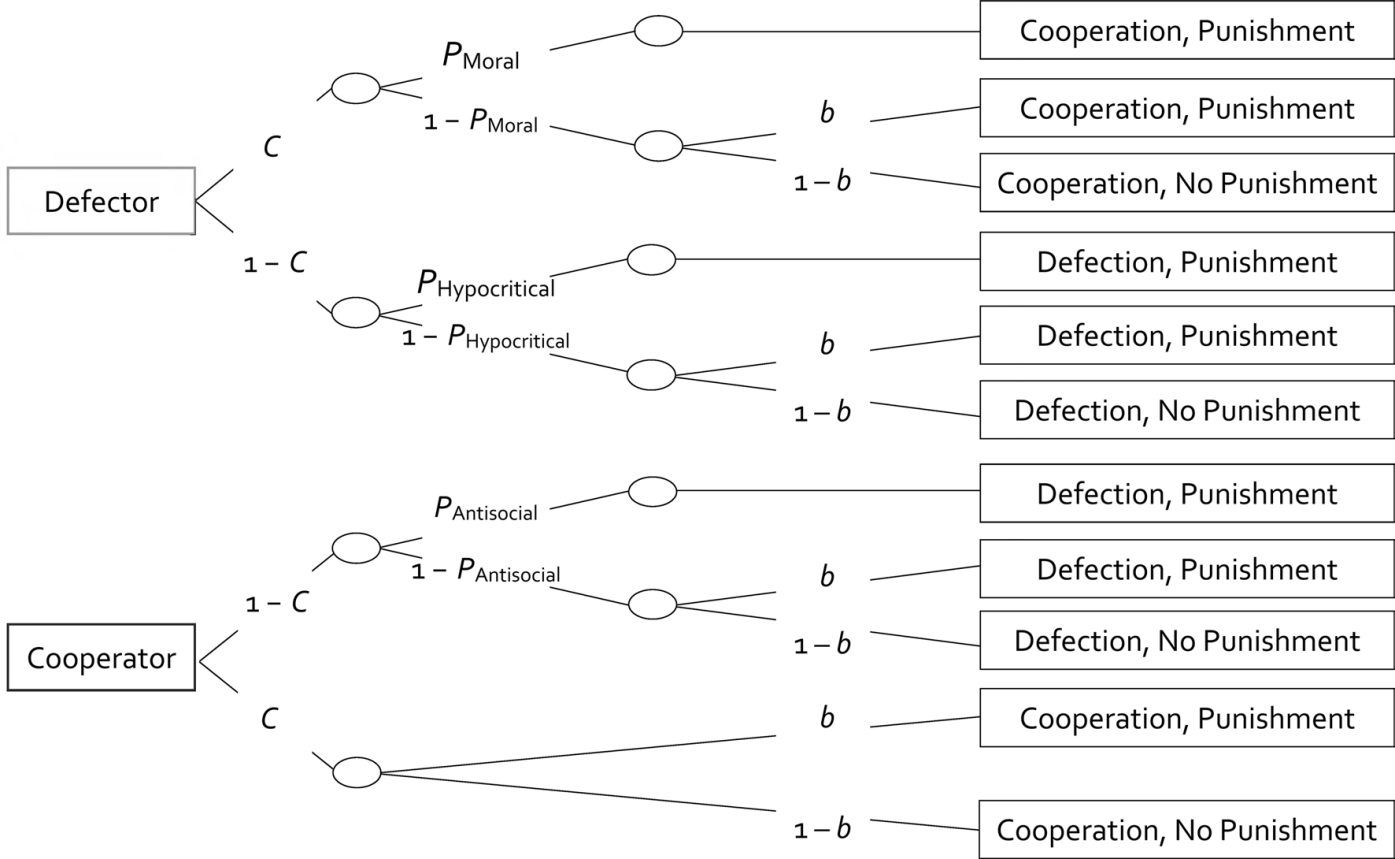
Multinomial cooperation-and-punishment model. Rectangles on the left represent the partner’s behavior. Rectangles on the right represent the participant’s behavior. Letters along the branches indicate the parameters of the model (*C* = cooperation, *P*_Moral_ = moral punishment of unilateral defection, *P*_Hypocritical_ = hypocritical punishment following mutual defection, *P*_Antisocial_ = antisocial punishment of unilateral cooperation; *b* = unspecific punishment bias).

Multinomial models have become increasingly popular as they allow to estimate the latent cognitive processes that underlie observable categorical behavioral data [e.g.,^42,43,56,57^]. The cooperation-and-punishment model used here has been successfully used to measure cooperation and punishment in previous studies^44–46^. It is illustrated in Fig. 3. The model incorporates two trees, one for the defecting partners and one for the cooperating partners. The first latent process specified in both trees is the participant’s propensity to cooperate which is assumed to be independent of the individual partner’s behavior that is revealed only after the participant’s decision. Therefore, the same parameter *C* can be used for both trees: Participants may choose to cooperate with probability *C* or to defect with probability 1–*C*. Depending on whether the partner cooperates or defects, distinct types of punishment may occur. If the participant’s cooperation is met with the partner’s defection, the participant may apply moral punishment with probability *P*_Moral_. Even if the participant does not apply moral punishment with probability 1 − *P*_Moral_, the participant may still punish the partner because of an unspecific punishment bias with probability *b*. With probability 1 − *b*, no punishment is applied. After the mutual defection of both players, hypocritical punishment may be applied with probability *P*_Hypocritical_. Even if no hypocritical punishment is applied with probability 1 − *P*_Hypocritical_, punishment may still occur due to the unspecific punishment bias with probability *b*. With probability 1 − *b*, no punishment is applied. If the participant’s defection mismatches with the cooperation of the partner, the participant may apply antisocial punishment with probability *P*_Antisocial_. If the participant does not apply antisocial punishment with probability 1–*P*_Antisocial_, punishment may still occur due to the unspecific punishment bias with probability *b*. With probability 1 − *b*, no punishment is applied. Mutual cooperation does not provide any specific reason to punish the partner. Any punishment in this case is therefore used to estimate the punishment bias *b* which reflects an unspecific tendency to punish the partner irrespective of the outcome of the interaction. To illustrate, if an emotion-centered processing focus induces feelings of frustration, this may well result in the indiscriminate punishment of partners irrespective of the outcomes of the Prisoner’s Dilemma game which is then reflected in the punishment bias *b*^46^*.* The model implies that this punishment bias has to be distinguished from types of punishment that discriminate between different partner behaviors in a parallel way to how response bias has to be distinguished from more specific responses in other decision-making models^58–62^. With probability 1–*b*, no punishment is applied.

### Results

When using multinomial models to test substantive hypotheses it is ideal to begin with a base model that fits the data. A multinomial model fits the data if the goodness-of-fit test assessing the discrepancy between the observed responses and the responses predicted by the model is non-significant, as indicated by a *p*-value larger than the α-level (usually 0.05). The corresponding goodness-of-fit statistic *G*^2^ is chi-square distributed with degrees of freedom indicated in parentheses. To analyze the present data, two sets of the trees of the multinomial cooperation-and-punishment model depicted in Fig. 3 are needed for the base model, one set for the cooperating-majority condition and one for the defecting-majority condition. This base model fit the data, *G*^2^(2) = 3.46, *p* = 0.177.

**Figure 4:**
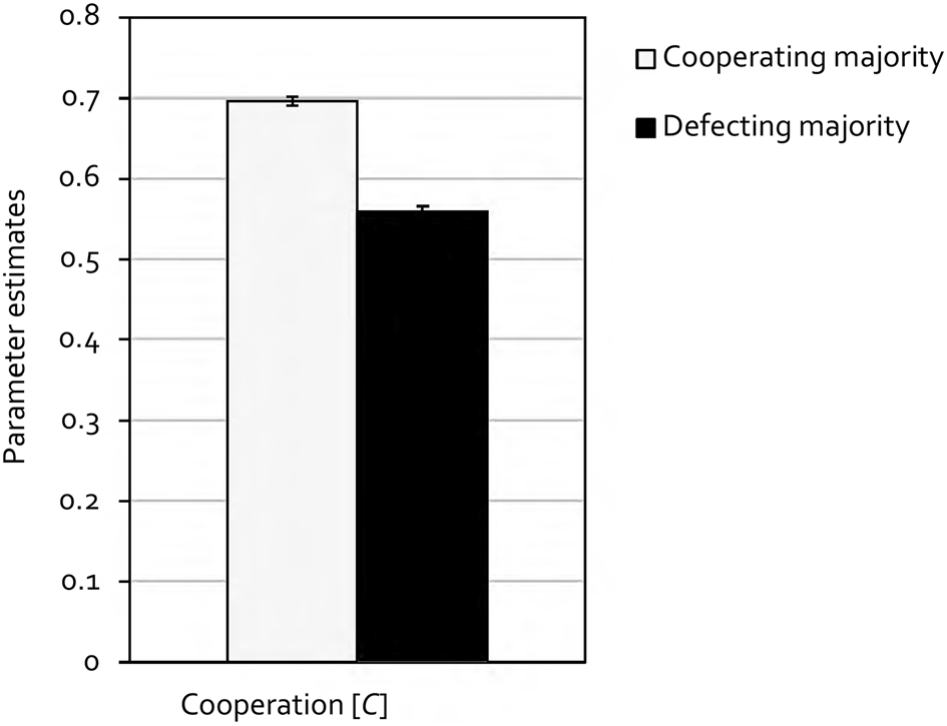
Estimates of the cooperation parameter *C* as a function of cooperation base rates in Experiment 1 (with partner punishment). In the cooperating-majority condition, partners cooperated in 60% of the trials and defected in the other 40%. In the defecting-majority condition, this ratio was reversed. Error bars represent standard errors.

Multinomial models allow hypothesis tests to be performed directly at the level of the parameters representing the cognitive processes assumed to underly observed behavior. For example, the hypothesis that the participants’ propensity to cooperate is significantly higher in the cooperating-majority condition than in the defecting-majority condition can be tested by restricting the *C* parameters of the two conditions to be equal. If this equality restriction significantly worsens the fit of the restricted model compared to the base model, as indicated by the Δ*G*^2^ statistic which is chi-square distributed with degrees of freedom displayed in parentheses, it can be concluded that the participants’ propensity to cooperate differs between the two conditions. Figure 4 displays the estimates of the cooperation parameter *C*. Cooperation was indeed significantly higher in the cooperating-majority condition than in the defecting-majority condition, Δ*G*^2^(1) = 272.57, *p* < 0.001, *w* = 0.14.

**Figure 5:**
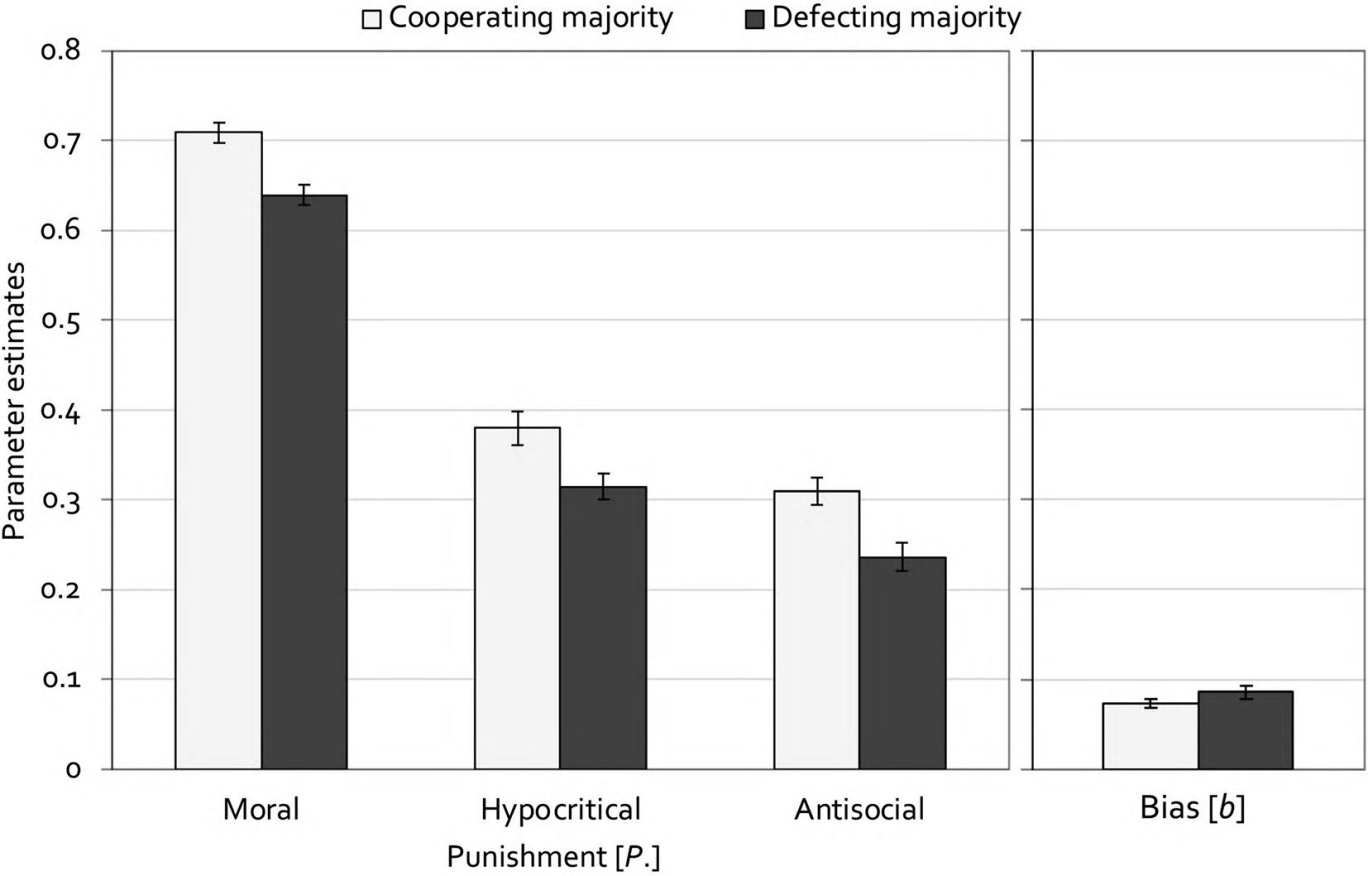
Estimates of the parameters representing moral, hypocritical and antisocial punishment (left panel) and the punishment bias (right panel) in Experiment 1 (with partner punishment). In the cooperating-majority condition, partners cooperated in 60% of the trials and defected in the other 40%. In the defecting-majority condition, this ratio was reversed. Error bars represent standard errors.

Estimates of the punishment parameters are shown in Fig. 5. In line with the conformity account, moral punishment was significantly higher in the cooperating-majority condition than in the defecting-majority condition, Δ*G*^2^(1) = 19.79, *p* < 0.001, *w* = 0.04. Also consistent with the conformity account, a high base rate of cooperation in comparison to defection led to an increase in hypocritical punishment, Δ*G*^2^(1) = 7.88, *p* = 0.005, *w* = 0.02. So far, the data seem compatible with the conformity account. However, participants were much more likely to use moral punishment to punish a deviation from the cooperating-majority group than to use antisocial punishment to punish a deviation from the defecting-majority group, Δ*G*^2^(1) = 557.29, *p* < 0.001, *w* = 0.20, which provides evidence against the assumption that punishment is exclusively determined by the goal to enforce conformity. Also, in direct opposition to the prediction of the conformity account, antisocial punishment was enhanced in the cooperating-majority condition compared to the defecting-majority condition, Δ*G*^2^(1) = 11.55, *p* = 0.001, *w* = 0.03. Finally, the punishment bias did not differ between the cooperating-majority condition and the defecting-majority condition, Δ*G*^2^(1) = 2.10, *p* = 0.147, *w* = 0.01.

### Discussion

The aim of the experiment was to test two accounts of punishment. If punishment serves to enforce conformity, then punishment should be directed at punishing *any* deviation from the majority and should therefore be affected by the proportion of cooperating and defecting partners. Specifically, moral punishment should be increased when cooperation is the dominant behavior whereas antisocial punishment should be increased when defection is the dominant behavior. In line with the conformity account, moral punishment was significantly higher in the cooperating-majority condition compared to the defecting-majority condition. In line with the idea that people punish to enforce conformity regardless of their own preferences^36^, hypocritical punishment was also higher in the cooperating-majority condition compared to the defecting-majority condition. However, if punishment were exclusively determined by the goal to enforce conformity, then the probability that participants use moral punishment to punish a deviation from the cooperating majority should be identical to the probability that they use antisocial punishment to punish a deviation from the defecting majority. This was clearly not the case. In addition, antisocial punishment was enhanced in the cooperating-majority condition compared to the defecting-majority condition which is also not compatible with the conformity account. In other words, these results clearly rule out that people punish what is uncommon without regard to the type of behavior that is shown. The fact that moral punishment was much more likely than antisocial punishment regardless of the proportion of cooperation and defection strongly suggests that, while punishment is affected by the base rates of cooperation and defection, punishment primarily serves to discourage defection^14^. Finally, it seems noteworthy that the punishment bias was not affected by the base-rate manipulation, suggesting that a high proportion of defection did not generally decrease the propensity to punish.

Similar to the punishment parameters, the probability of cooperation was significantly higher in the cooperating-majority condition than in the defecting-majority condition. This is in line with a bulk of studies reporting how participants condition their own cooperation on the perceived or proclaimed cooperation rates of others^16,19–21,23^. Interestingly, while being clearly influenced by the prevailing cooperation rates, participants’ propensity to cooperate still exceeded the base rate of cooperation in both the cooperating-majority condition and the defecting-majority condition: When partners cooperated in 60% of the trials in the cooperating-majority condition, participants cooperated in 70% of the trials, whereas when partners cooperated in 40% of the trials in the defecting-majority condition, participants nevertheless cooperated in 56% of the trials.

Cooperation rates may have been elevated in Experiment 1 because the partners reliably punished the unilateral defection of the participants and thereby discouraged defection. This could potentially also explain why moral punishment remained at a high level in the cooperating-majority condition as well as the defecting-majority condition in that it seems conceivable that participants may have followed the example of their partners when deciding to apply moral punishment [cf.^37,63–65^]. It thus is necessary to test how the proportion of cooperation and defection affects moral punishment when participants cannot base their own punishment decisions on the example set by the partners. Therefore, we tested in Experiment 2 how the proportion of cooperating and defecting partners affects moral punishment when punishment is unilaterally available to the participants but not to the partners, as in previous experiments^44,66,67^. If the effects of the base-rate manipulation are independent of the presence or absence of partner punishment, the pattern of results from Experiment 1 should be replicated. To the degree that the effects of the base rate manipulation depend on the presence or absence of the partners’ moral punishment, the effects should differ between Experiments 1 and 2.

## Experiment 2

### Method

Parallel to Experiment 1, we aimed at recruiting about 500 valid data sets with the help of the online panel provider *mingle.* Of those participants who had started the game, 54 data files had to be excluded because the participants did not complete the experiment; 48 data files had to be excluded due to double participation. The final sample consisted of *N* = 495 participants (209 female, 284 male, 2 non-binary) aged 18–90 years with a mean age of 49 (*SD* = 16) years. The slightly smaller sample size relative to that of Experiment 1 (*n* = 544) did not substantially affect the sensitivity of the statistical tests. It was still possible to detect effects of *w* = 0.03 with a statistical power of 1 – β = 0.95 at an α level of 0.05 when comparing the cooperation and punishment parameters between the cooperating-majority condition (*n* = 250) and the defecting-majority condition (*n* = 245)^47^.

Materials and procedure were identical to those of Experiment 1 with the exception that the punishment option was unilaterally available to the participants, implying that the partners did not punish participants’ defection. Participants therefore only received feedback about their own punishment decision and its effect on the partner’s account balance. The average final account balance was 275 (*SD* = 100) points.

### Results

**Figure 6:**
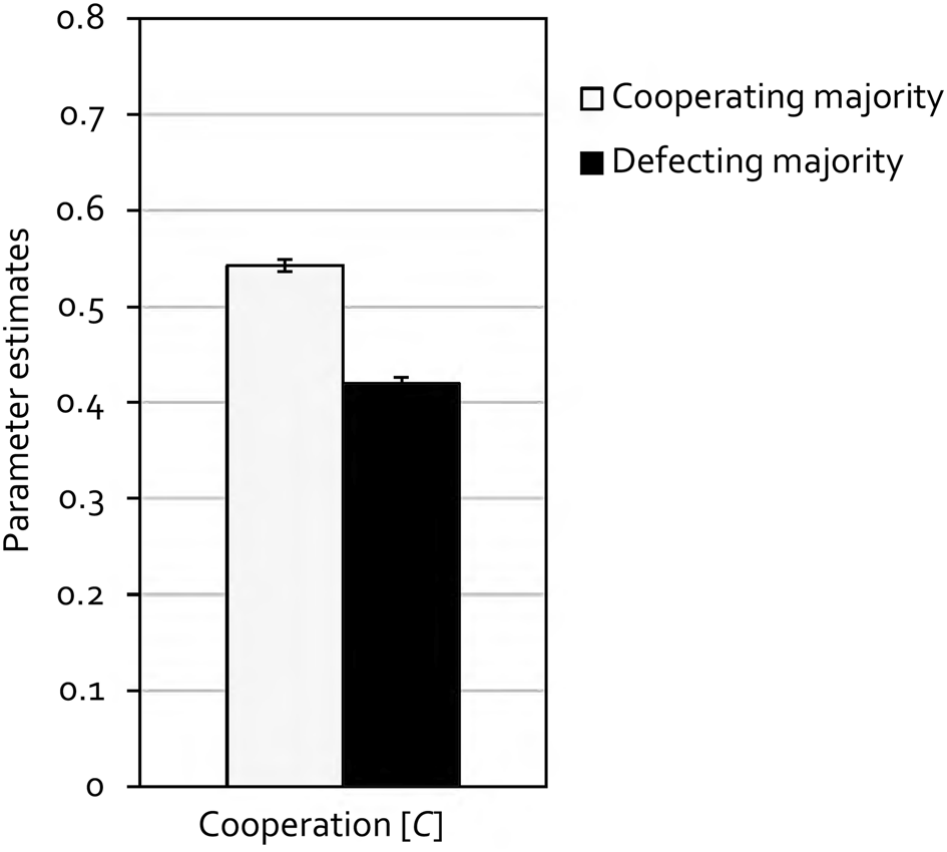
Estimates of the cooperation parameter *C* as a function of cooperation base rates in Experiment 2 (without partner punishment). In the cooperating-majority condition, partners cooperated in 60% of the trials and defected in the other 40%. In the defecting-majority condition, this ratio was reversed. Error bars represent standard errors.

As in Experiment 1, the data were analyzed using the multinomial cooperation-and-punishment model (see Fig. 3). The goodness-of-fit test showed that the base model provided a good fit to the data, *G*^2^(2) = 0.40, *p* = 0.819. The estimates of the cooperation parameter *C* are shown in Fig. 6. Replicating the results of Experiment 1, cooperation was significantly higher in the cooperating-majority condition in comparison to the defecting-majority condition, Δ*G*^2^(1) = 188.35, *p* < 0.001, *w* = 0.12.

**Figure 7:**
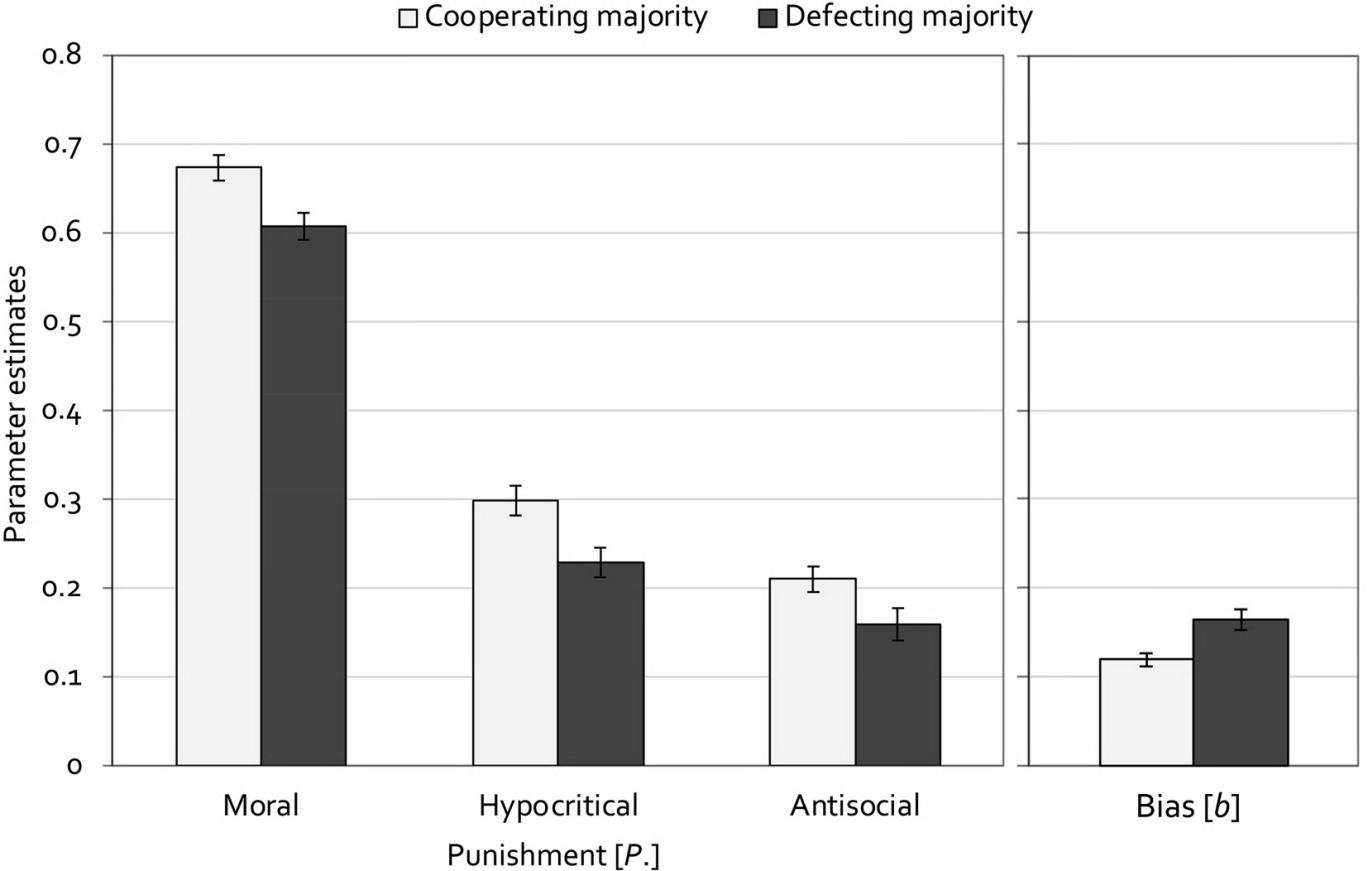
Estimates of the parameters representing moral, hypocritical, and antisocial punishment (left panel) and the punishment bias (right panel) in Experiment 2 (without partner punishment). In the cooperating-majority condition, partners cooperated in 60% of the trials and defected in the other 40%. In the defecting-majority condition, this ratio was reversed. Error bars represent standard errors.

Figure 7 displays the estimates of the punishment parameters (left panel) and the punishment bias (right panel). In line with Experiment 1, moral punishment was significantly higher in the cooperating-majority condition than in the defecting-majority condition, Δ*G*^2^(1) = 10.01, *p* = 0.002, *w* = 0.03. Also consistent with Experiment 1, a high base rate of cooperation in comparison to defection led to an increase in hypocritical punishment, Δ*G*^2^(1) = 8.70, *p* = 0.003, *w* = 0.03. Further replicating Experiment 1 and in direct opposition to the prediction of the conformity account, moral punishment in the cooperating-majority group was much more likely than antisocial punishment in the defecting-majority group, Δ*G*^2^(1) = 486.20, *p* < 0.001, *w* = 0.20, which is evidence against the assumption that these types of punishment are exclusively determined by the goal to enforce conformity. Parallel to the results of Experiment 1, and further disconfirming the conformity account, antisocial punishment was enhanced in the cooperating-majority condition compared to the defecting-majority condition, Δ*G*^2^(1) = 4.87, *p* = 0.027, *w* = 0.02. Finally, the punishment bias was significantly higher in the defecting-majority condition than in the cooperating-majority condition, Δ*G*^2^(1) = 11.46, *p* = 0.001, *w* = 0.03.

### Discussion

The aim of Experiment 2 was to test whether the effects of Experiment 1 can be replicated when partners do not morally punish defection. Replicating the main findings of Experiment 1, moral, hypocritical and antisocial punishment were significantly higher in the cooperating-majority condition in comparison to the defecting-majority condition in Experiment 2. While the effects of the base-rate manipulation on moral and hypocritical punishment are partly in line with the conformity account, the effect on antisocial punishment is in direct opposition to what the conformity account implies, as is the fact that moral punishment in the cooperating-majority group was much more likely than antisocial punishment in the defecting-majority group. This necessarily leads to the conclusion that people do not punish behavior only because it deviates from what the majority does. Interestingly, moral punishment rates still remained at a high level even though, in contrast to Experiment 1, participants could not follow their partners’ example when deciding whether to use moral punishment. This supports the conclusion that when applying moral punishment people are not merely conforming to the observed punishment behavior of their partners. Instead, there seems to be an intrinsic motive for punishing defection. The present results thereby nicely fit with the recently proposed moral preference hypothesis according to which costly punishment of defection is driven by an internalized preference to act in a way that is typically considered moral^68,69^. Other than in Experiment 1, the punishment bias was increased in the defecting-majority condition in Experiment 2. This suggests that when mainly interacting with defecting partners, participants tend to randomly punish their partners more frequently, possibly as a way to vent frustration about the high prevalence of defection. As in Experiment 1, it can be concluded that there was no general reluctance to punish in the defecting-majority condition.

The effect of the base-rate manipulation on the participants’ own inclination to cooperate was replicated in Experiment 2. Moreover, when cooperation was not enforced by moral punishment, participants’ own cooperation rates aligned more closely with the manipulated base rates than participants’ cooperation rates in Experiment 1. This points to a conformist motive behind cooperation, in line with the previous literature^16,21,23^.

## General discussion

The moral punishment of defection is integral to enforcing and maintaining cooperation in the light of the free-rider problem e.g.,^8,13^. It is therefore important to understand what drives people to accept the costs associated with punishing others. If punishment primarily serves to discourage defection^14^, people should use the punishment option primarily to morally punish unilateral defection while antisocial punishment should occur with a comparatively smaller probability regardless of whether the majority of the partners cooperates or defects. If punishment primarily serves to enforce conformity^15–18,36^, people should punish all behaviors that do not conform to what the majority does regardless of the specific type of behavior in question. Both accounts predict that people will primarily use moral punishment when most people cooperate. However, the conformity account makes the unique prediction that moral punishment should become less prevalent when most people defect. The present study followed a previous study by Li et al.^37^ who found that moral punishment indeed decreases with decreasing cooperation rates. A limitation of the previous study was that participants conditioned their responses on instructed hypothetical base rates of cooperative behavior without experiencing them directly. In the present study, we used a Prisoner’s Dilemma game with a costly punishment option and manipulated whether the participants’ partners cooperated or defected in the majority (60%) of trials. In line with the study by Li et al.^37^, we consistently found across two experiments that moral punishment was more prevalent in the cooperating-majority condition than in the defecting-majority condition. Extending the previous study, we found across both experiments that hypocritical punishment was also more prevalent when the base rate of cooperation was high compared to when it was low. This pattern is consistent with the idea that people may enforce conformity with the majority even when they do not share the preferences of the majority^36^.

So far, the results seem to support the conformity account. However, there are several aspects of the results that are inconsistent with this account. First, moral punishment of defection in the cooperating-majority group was much more likely than antisocial punishment of cooperation in the defecting-majority group which is inconsistent with the assumption that these types of punishment are exclusively determined by the goal to enforce conformity. If that were the case, then the probability of antisocial punishment in the defecting-majority condition should be as high as the probability of moral punishment in the cooperating-majority condition. This prediction is clearly contradicted by the data we observed. Another important prediction of the conformity account is that people should be more likely to use antisocial punishment to punish cooperation in the defecting-majority condition than in the cooperating-majority condition. However, antisocial punishment was lower in the defecting-majority condition than in the cooperating-majority condition, in direct opposition to the prediction of the conformity account.

Overall, the results are thus most compatible with an integrative account according to which people primarily use punishment to discourage defection^14^ but still adjust the punishment to the perceived cooperation levels. A high prevalence of cooperation is often believed to create or strengthen a cooperative norm^22,23,70^. Therefore, defection in a cooperative environment may be perceived as being more deviant and thus more deserving of punishment than defection in an environment in which defection is common^37,71^. Hypocritical punishment may be used to make up for one’s own failure to adhere to the cooperative norm as it has been observed that people tend to use punishment to feign sincere support of the majority group behavior despite their actual disapproval^36^. Antisocial punishment may be assumed to be driven by an opposition to the normative pressure towards cooperation that is not shared. For instance, antisocial punishment has often been attributed to an aversion to morally superior “do-gooders”^31,33–35^. People may use antisocial punishment as a retaliation for the embarrassment evoked by one’s unilateral defection. When cooperation is more prevalent, the embarrassment that is caused by the norm violation could well be amplified, causing a stronger urge to harm or devaluate the opponent for causing the embarrassment. In fact, increased levels of do-gooder derogation have been reported when the perceived number of people belonging to the morally superior group was high because a strong conformist pressure created a stronger threat to one’s moral identity ^72,73^. It thus is psychologically plausible that antisocial punishment increases rather than decreases with a strong normative pressure towards cooperation as it may reflect a direct opposition towards cooperation.

Given that the present results suggest that high cooperation levels lead to more antisocial punishment, the question arises as to why the prevalence of antisocial punishment is often negatively related to the prevalence of cooperation in cross-cultural comparisons^28,31^ in which participants from societies with low cooperation rates usually experience more antisocial punishment. Here it must be kept in mind that such findings are only correlational and the low cooperation levels might be a consequence of the detrimental effect of antisocial punishment on cooperation instead of the cause for the high antisocial punishment. In the present study, we used an experimental manipulation of the proportion of cooperation and defection to identify its effects on the different types of punishment without having to second-guess the direction of the effects. It also seems striking that most evidence in favor of the conformity account of costly punishment comes from the Public Goods game that examines cooperation within larger groups^16,17^, but see^23^. It thus seems conceivable that the requirement to find a balance between individual and collective interests in larger group settings may create stronger conformist pressures than the dyadic interactions in the Prisoner’s Dilemma game.

Finally, it seems noteworthy that a conformity effect was not only observed with respect to punishment but also with respect to cooperation. Participants’ willingness to cooperate was clearly affected by whether the majority of the partners cooperated or defected. This is in line with a bulk of studies on how participants condition their cooperation on perceived or proclaimed cooperation rates of others^16,19–23^. Interestingly, cooperation rates clearly exceeded the manipulated base rate when the partners applied moral punishment to discourage defection (Experiment 1). Without partner punishment (Experiment 2), participants lacked an economic incentive to cooperate. As a result, the participants’ propensity to cooperate aligned more closely with the manipulated base rates which therefore points to a conformist motive behind cooperation.

The aim of the present experiments was to test whether costly punishment is affected by the prevalence of cooperation. By varying the cooperation rates of simulated interaction partners in a between-groups design we were able to experimentally manipulate the base rates of cooperation and defection while maintaining experimental control over extraneous factors that may otherwise influence the players’ behaviors. This approach differs from what is common practice in Experimental Economics but conforms to research traditions in Experimental Psychology [e.g.,^16,48,50,52,54^]. In this context, two observations seem worth noting. First, participants readily cooperated with, and even punished, their partners even though this implied sacrificing some of their own money. Second, the punishment rates observed in the highly controlled experiments presented here are comparable to the punishment rates reported in studies using real interaction partners [e.g.,^12^,^27^]. Taken together, these observations suggest that the present experimental paradigm reliably activated mechanisms of social interactions. Still, it is of course an intriguing avenue for future research to test whether the present conclusions generalize to different settings in which, for instance, participants interact in human dyads.

### Conclusion

Do we punish others for failing to conform to the majority irrespective of the specific type of behavior in question? The present results clearly demonstrate that people do not punish a specific behavior only because it is uncommon. Regardless of the prevalence of cooperation or defection, participants primarily used moral punishment to express their disapproval of a partner’s unilateral defection. This indicates that punishment is primarily used to discourage defection and not to enforce blind conformity with the majority. Nevertheless, there were several ways in which participants’ behaviors were sensitive to the proportion of cooperation and defection they experienced. The present results corroborate previous findings [cf.^37^] suggesting that moral punishment increases with the proportion of cooperating partners in the Prisoner’s Dilemma game. In other words, defecting behavior that deviates from what the majority does is punished more. The same was found for hypocritical punishment. Nevertheless, moral punishment of deviations from a cooperating majority was much higher than antisocial punishment of deviations from a defecting majority which should not be the case if these types of punishment were exclusively determined by the goal to enforce conformity. Furthermore, antisocial punishment was increased when the prevalence of cooperation was high which suggests that antisocial punishment increases with the perceived pressure towards cooperation. Punishment is thus sensitive to the rates of cooperation and defection but, overall, the results are inconsistent with the idea that punishment primarily, let alone exclusively, serves to enforce conformity.

### Ethics approval and consent to participate

The study was conducted in accordance with the guidelines laid down in the Declaration of Helsinki and by the German Research Foundation (DFG) including confidentiality of data and personal conduct. Informed consent was obtained prior to participation. For the noninvasive, purely behavioral research reported in the present series of experiments which carried no risk for the participants, a formal approval by the institution’s ethical board is not legally required in Germany (see: https://www.dfg.de/en/research_funding/faq/faq_humanities_social_science/index.html).

## Data Availability

We provide the data used in our analyses via the Open Science Framework. The data is publicly available at https://osf.io/fycg3/.

## References

[1] Kollock P (1998). Social dilemmas: The anatomy of cooperation. Annu. Rev. Sociol..

[2] Fehr E, Fischbacher U (2004). Social norms and human cooperation. Trends Cognit. Sci..

[3] Andreoni J (1995). Cooperation in public-goods experiments: Kindness or confusion?. Am. Econ. Rev..

[4] Nowak MA (2006). Five rules for the evolution of cooperation. Science.

[5] Boyd R, Richerson PJ (1992). Punishment allows the evolution of cooperation (or anything else) in sizable groups. Ethol. Sociobiol..

[6] Rapoport A, Chammah AM (1965). Prisoner’s Dilemma: A Study in Conflict and Cooperation.

[7] Chen X, Szolnoki A, Perc M (2015). Competition and cooperation among different punishing strategies in the spatial public goods game. Phys. Rev. E..

[8] Ostrom E, Walker J, Gardner R (1992). Covenants with and without a sword: Self-governance is possible. Am. Polit. Sci. Rev..

[9] Boyd R, Gintis H, Bowles S, Richerson PJ (2003). The evolution of altruistic punishment. Proc. Natl. Acad. Sci..

[10] Hua S, Liu L (2023). Facilitating the evolution of cooperation through altruistic punishment with adaptive feedback. Chaos Solitons Fractals.

[11] Fehr E, Gächter S (2000). Cooperation and punishment in public goods experiments. Am. Econ. Rev..

[12] Falk A, Fehr E, Fischbacher U (2005). Driving forces behind informal sanctions. Econometrica.

[13] Fehr E, Gächter S (2002). Altruistic punishment in humans. Nature.

[14] Bone J, Silva AS, Raihani NJ (2014). Defectors, not norm violators, are punished by third-parties. Biol. Lett..

[15] Horne C (2009). The Rewards of Punishment: A Relational Theory of Norm Enforcement.

[16] Irwin K, Horne C (2013). A normative explanation of antisocial punishment. Soc. Sci. Res..

[17] Horne C, Irwin K (2016). Metanorms and antisocial punishment. Soc. Influ..

[18] Carpenter JP, Matthews PH (2012). Norm enforcement: Anger, indignation, or reciprocity?. J. Eur. Econ. Assoc..

[19] Fischbacher U, Gächter S, Fehr E (2001). Are people conditionally cooperative? Evidence from a public goods experiment. Econ. Lett..

[20] Kocher MG, Cherry T, Kroll S, Netzer RJ, Sutter M (2008). Conditional cooperation on three continents. Econ. Lett..

[21] Chaudhuri A (2011). Sustaining cooperation in laboratory public goods experiments: A selective survey of the literature. Exp. Econ..

[22] Fowler JH, Christakis NA (2010). Cooperative behavior cascades in human social networks. Proc. Natl. Acad. Sci..

[23] Engel C, Kube S, Kurschilgen M (2021). Managing expectations: How selective information affects cooperation and punishment in social dilemma games. J. Econ. Behav. Organ..

[24] Clutton-Brock TH, Parker GA (1995). Punishment in animal societies. Nature.

[25] Gurerk O, Irlenbusch B, Rockenbach B (2006). The competitive advantage of sanctioning institutions. Science.

[26] Przepiorka W, Diekmann A (2013). Individual heterogeneity and costly punishment: A volunteer's dilemma. Proc. R. Soc. B. Biol. Sci..

[27] Carpenter JP (2007). The demand for punishment. J. Econ. Behav. Organ..

[28] Henrich J (2006). Costly punishment across human societies. Science.

[29] Cinyabuguma M, Page T, Putterman L (2006). Can second-order punishment deter perverse punishment?. Exp. Econ..

[30] Pfattheicher S, Keller J, Knezevic G (2017). Sadism, the intuitive system, and antisocial punishment in the public goods game. Pers. Soc. Psychol. Bull..

[31] Herrmann B, Thoni C, Gachter S (2008). Antisocial punishment across societies. Science.

[32] Sylwester K, Herrmann B, Bryson JJ (2013). Homo homini lupus? Explaining antisocial punishment. J. Neurosci. Psychol. Econ..

[33] Monin B (2007). Holier than me? Threatening social comparison in the moral domain. Int. Rev. Soc. Psychol..

[34] Gächter S, Herrmann B (2009). Reciprocity, culture and human cooperation: Previous insights and a new cross-cultural experiment. Philos. Trans. R. Soc. Lond. B Biol. Sci..

[35] Pleasant A, Barclay P (2018). Why hate the good guy? Antisocial punishment of high cooperators is greater when people compete to be chosen. Psychol. Sci..

[36] Willer R, Kuwabara K, Macy MW (2009). The false enforcement of unpopular norms. Am. J. Sociol..

[37] Li X, Molleman L, van Dolder D (2021). Do descriptive social norms drive peer punishment? Conditional punishment strategies and their impact on cooperation. Evol. Hum. Behav..

[38] Erdfelder E (2009). Multinomial processing tree models: A review of the literature. Z. für Psychologie/J. Psychol..

[39] Schmidt O, Erdfelder E, Heck DW (2023). How to develop, test, and extend multinomial processing tree models: A tutorial. Psychol. Methods.

[40] Moshagen M (2010). multiTree: A computer program for the analysis of multinomial processing tree models. Behav. Res. Methods.

[41] Castela M, Kellen D, Erdfelder E, Hilbig BE (2014). The impact of subjective recognition experiences on recognition heuristic use: A multinomial processing tree approach. Psychon. Bull. Rev..

[42] Klauer KC, Stahl C, Erdfelder E (2007). The abstract selection task: New data and an almost comprehensive model. J. Exp. Psychol. Learn. Mem. Cogn..

[43] Gawronski B, Armstrong J, Conway P, Friesdorf R, Hütter M (2017). Consequences, norms, and generalized inaction in moral dilemmas: The CNI model of moral decision-making. J. Pers. Soc. Psychol..

[44] Mieth L, Buchner A, Bell R (2021). Moral labels increase cooperation and costly punishment in a Prisoner’s Dilemma game with punishment option. Sci. Rep..

[45] Mieth L, Buchner A, Bell R (2021). Cognitive load decreases cooperation and moral punishment in a Prisoner’s Dilemma game with punishment option. Sci. Rep..

[46] Philippsen A, Mieth L, Buchner A, Bell R (2023). Communicating emotions, but not expressing them privately, reduces moral punishment in a Prisoner’s Dilemma game. Sci. Rep..

[47] Faul F, Erdfelder E, Lang A-G, Buchner A (2007). G* Power 3: A flexible statistical power analysis program for the social, behavioral, and biomedical sciences. Behav. Res. Methods.

[48] Parks CD, Stone AB (2010). The desire to expel unselfish members from the group. J. Pers. Soc. Psychol..

[49] Bell R, Mieth L, Buchner A (2017). Separating conditional and unconditional cooperation in a sequential Prisoner’s Dilemma game. PLoS ONE.

[50] Wang L, Zheng J, Meng L, Lu Q, Ma Q (2016). Ingroup favoritism or the black sheep effect: Perceived intentions modulate subjective responses to aggressive interactions. Neurosci. Res..

[51] Sanfey AG, Rilling JK, Aronson JA, Nystrom LE, Cohen JD (2003). The neural basis of economic decision-making in the ultimatum game. Science.

[52] Barclay P (2008). Enhanced recognition of defectors depends on their rarity. Cognition.

[53] Bell R, Buchner A, Musch J (2010). Enhanced old–new recognition and source memory for faces of cooperators and defectors in a social-dilemma game. Cognition.

[54] Volstorf J, Rieskamp J, Stevens JR (2011). The good, the bad, and the rare: Memory for partners in social interactions. PLoS ONE.

[55] Ma DS, Correll J, Wittenbrink B (2015). The Chicago face database: A free stimulus set of faces and norming data. Behav. Res. Methods.

[56] Riefer DM, Batchelder WH (1988). Multinomial modeling and the measurement of cognitive processes. Psychol. Rev..

[57] Kroneisen M, Steghaus S (2021). The influence of decision time on sensitivity for consequences, moral norms, and preferences for inaction: Time, moral judgments, and the CNI model. J. Behav. Decis. Mak..

[58] Bayen UJ, Murnane K, Erdfelder E (1996). Source discrimination, item detection, and multinomial models of source monitoring. J. Exp. Psychol. Learn. Mem. Cogn..

[59] Buchner A, Erdfelder E, Vaterrodt-Plünnecke B (1995). Toward unbiased measurement of conscious and unconscious memory processes within the process dissociation framework. J. Exp. Psychol. Gen..

[60] Menne NM, Winter K, Bell R, Buchner A (2022). A validation of the two-high threshold eyewitness identification model by reanalyzing published data. Sci. Rep..

[61] Batchelder WH, Riefer DM (1990). Multinomial processing models of source monitoring. Psychol. Rev..

[62] Erdfelder E, Cüpper L, Auer T-S, Undorf M (2007). The four-states model of memory retrieval experiences. Z. Psychol./J Psychol..

[63] Son J-Y, Bhandari A, FeldmanHall O (2019). Crowdsourcing punishment: Individuals reference group preferences to inform their own punitive decisions. Sci. Rep..

[64] FeldmanHall O, Otto AR, Phelps EA (2018). Learning moral values: Another’s desire to punish enhances one’s own punitive behavior. J. Exp. Psychol. Gen..

[65] Suleiman R, Samid Y (2021). Punishment strategies across societies: Conventional wisdoms reconsidered. Games.

[66] Mieth L, Bell R, Buchner A (2016). Facial likability and smiling enhance cooperation, but have no direct effect on moralistic punishment. J. Exp. Psychol..

[67] Mieth L, Buchner A, Bell R (2017). Effects of gender on costly punishment. J. Behav. Decis. Mak..

[68] Capraro V, Jordan JJ, Tappin BM (2021). Does observability amplify sensitivity to moral frames? Evaluating a reputation-based account of moral preferences. J. Exp. Soc. Psychol..

[69] Capraro V, Perc M (2021). Mathematical foundations of moral preferences. J. R. Soc. Interface.

[70] Peysakhovich A, Rand DG (2016). Habits of virtue: Creating norms of cooperation and defection in the laboratory. Manag. Sci..

[71] Lindström B, Jangard S, Selbing I, Olsson A (2018). The role of a “common is moral” heuristic in the stability and change of moral norms. J. Exp. Psychol. Gen..

[72] Minson JA, Monin B (2012). Do-gooder derogation: Disparaging morally motivated minorities to defuse anticipated reproach. Soc. Psychol. Personal. Sci..

[73] Loughnan, S. & Piazza, J. in *Atlas of moral psychology* (eds Kurt Gray & Jesse Graham) 165–174 (2018).

